# Systematic engineering of the central metabolism in *Escherichia coli* for effective production of *n-*butanol

**DOI:** 10.1186/s13068-016-0467-4

**Published:** 2016-03-18

**Authors:** Mukesh Saini, Si-Yu Li, Ze Win Wang, Chung-Jen Chiang, Yun-Peng Chao

**Affiliations:** Department of Chemical Engineering, Feng Chia University, 100 Wenhwa Road, Taichung, 40724 Taiwan Republic of China; Department of Chemical Engineering, National Chung Hsing University, Taichung, 402 Taiwan Republic of China; Department of Medical Laboratory Science and Biotechnology, China Medical University, No. 91, Hsueh-Shih Road, Taichung, 40402 Taiwan Republic of China; Department of Health and Nutrition Biotechnology, Asia University, Taichung, 41354 Taiwan Republic of China; Department of Medical Research, China Medical University Hospital, Taichung, 40447 Taiwan Republic of China

**Keywords:** *n-*Butanol, The redox state, Metabolic engineering, *Escherichia coli*

## Abstract

**Background:**

Microbes have been extensively explored for production of environment-friendly fuels and chemicals. The microbial fermentation pathways leading to these commodities usually involve many redox reactions. This makes the fermentative production of highly reduced products challenging, because there is a limited NADH output from glucose catabolism. Microbial production of *n-*butanol apparently represents one typical example.

**Results:**

In this study, we addressed the issue by adjustment of the intracellular redox state in *Escherichia coli*. This was initiated with strain BuT-8 which carries the clostridial CoA-dependent synthetic pathway. Three metabolite nodes in the central metabolism of the strain were targeted for engineering. First, the pyruvate node was manipulated by enhancement of pyruvate decarboxylation in the oxidative pathway. Subsequently, the pentose phosphate (PP) pathway was amplified at the glucose-6-phosphate (G6P) node. The pathway for G6P isomerization was further blocked to force the glycolytic flux through the PP pathway. It resulted in a growth defect, and the cell growth was later recovered by limiting the tricarboxylic acid cycle at the acetyl-CoA node. Finally, the resulting strain exhibited a high NADH level and enabled production of 6.1 g/L *n-*butanol with a yield of 0.31 g/g-glucose and a productivity of 0.21 g/L/h.

**Conclusions:**

The production efficiency of fermentative products in microbes strongly depends on the intracellular redox state. This work illustrates the flexibility of pyruvate, G6P, and acetyl-CoA nodes at the junction of the central metabolism for engineering. In principle, high production of reduced products of interest can be achieved by individual or coordinated modulation of these metabolite nodes.

**Electronic supplementary material:**

The online version of this article (doi:10.1186/s13068-016-0467-4) contains supplementary material, which is available to authorized users.

## Background

Our daily life is tightly linked to the petroleum-based industries. However, the rising price, the insecure supply, and the environmental concern of fossil fuels have currently overshadowed these industries. Consequently, it provokes the demand for renewable and environment-friendly fuels and chemicals [1]. The bioprocess production of these chemical commodities appears to be appealing [2, 3]. Bio-based fuels and chemicals of interest are generally fermentative products of living microbes. The microbial fermentation pathways involve many redox reactions, which usually require NADH and NAD^+^ as cofactors. With NAD^+^ as an electron acceptor, the oxidation of sugars produces NADH. NAD^+^ is regenerated when intermediate metabolites in the sugar catabolism are subsequently reduced at the expense of NADH. The result of the reductive reactions usually leads to production of ethanol, lactate, and succinate as exemplified in fermentative *Escherichia coli* [4]. Therefore, maintaining the redox balance of NADH and NAD^+^ is a key to ensure the continued operation of cellular metabolism under the fermentative condition.

Fermentative production of *n-*butanol in *Clostridium* species is a well-known bioprocess [5]. The fermentation process mainly consists of the acidogenesis and the solventogenesis phase [6]. In the acidogenesis stage, the growing *Clostridium* species ferment glucose to mainly produce acetate and butyrate. Upon reaching the stationary growth phase, the bacteria re-assimilate these organic acids, while acetone, *n*-butanol, and ethanol are produced as end products. In view of the clostridial synthetic pathway, the direct synthesis of *n*-butanol from glucose can cause the NADH/NAD^+^ redox imbalance because more NADH is required in the synthetic pathway than that generated in the glycolytic pathway. This may explain why the synthesis of *n*-butanol in *Clostridium* species proceeds in two stages.

*n*-Butanol is an alternative fuel of potential because its property is superior to ethanol in terms of the energy density, the vapor pressure, and hygroscopicity [7]. Moreover, *n*-butanol can be used for the transportation fuel after blended with gasoline at any concentrations and transported with the existing pipeline infrastructure [8]. These merits make microbial production of *n*-butanol industrially attractive. Many approaches have been proposed for production of *n*-butanol in a variety of surrogate strains [9–12]. However, these attempts are generally discouraged by their low *n*-butanol titer. Recognized as the biotechnology workhorse, *E. coli* has been commonly employed to produce value-added chemicals and biofuels [13, 14]. Production of *n*-butanol in *E. coli* is proven feasible after introduction of the clostridial synthetic pathway into the strain [9, 15, 16]. In addition, the production titer is improved by manipulating pyruvate dehydrogenase (PDH) and formate dehydrogenase (FDH) in glycolysis to increase the NADH availability in the cell [15, 17, 18]. Recently, we have proposed a new production platform based on two strains: a butyrate-conversion strain and a butyrate-producing strain [19]. The dual-culture system rebuilds a redox-balanced synthetic pathway, which enables effective production of *n-*butanol.

**Fig. 1:**
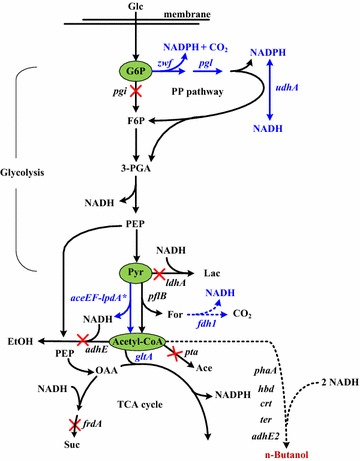
The central metabolic pathways leading to *n-*butanol in *E. coli*. The dotted lines denote the heterologous pathways. The CoA-dependent synthetic pathway of *n-*butanol is composed of heterologous *phaA*, *hbd*, *crt*, *ter*, and *adhE2* genes as shown. Three metabolite nodes including G6P, pyruvate, and acetyl-CoA are targeted for engineering and marked. The genes involved in the metabolic pathways: *aceEF*-lpdA*, pyruvate dehydrogenase complex; *adhE*, aldehyde-alcohol dehydrogenase; *adhE2*, butyraldehyde-butanol dehydrogenase; *crt*, crotonese; *gltA*, citrate synthase; *hbd*, 3-hydroxybutyryl-CoA dehydrogenase; *ldhA*, lactate dehydrogenase; *fdh1*, formate dehydrogenase; *frdA*, subunit of fumarate reductase; *pflB*, pyruvate formate-lyase; *pgi*, phosphoglucose isomerase; *pgl*, lactonase; *phaA*, acetoacetyl-CoA thiolase; *pta*, phosphate acetyltransferase; *ter*, trans-enoyl-CoA reductase; *udhA*, transhydrogenase; *zwf*, glucose-6-phosphate dehydrogenase. The deleted genes are indicated by “X”. Abbreviations: *Ace* acetate; *EtOH* ethanol; *F6P* fructose-6-phosphate; *Lac* lactate; *For* formate; *G6P* glucose-6-phosphate; *Glc* glucose; *OAA* oxaloacetate; *PEP* phosphoenolpyruvate; *3-PGA* 3-phosphoglyceraldehyde; *Pyr* pyruvate; *Suc* succinate

Microbial production of a highly reduced fermentation product such as *n-*butanol remains challenging because there is a limited NADH output from glucose catabolism. This issue was addressed by rerouting the central metabolic pathways in *E. coli*. The NADH availability is manifested by the interplay of glycolysis, the pentose phosphate (PP) pathway, and the tricarboxylic acid (TCA) cycle involved in central metabolism (Fig. 1). In addition to PDH and FDH, the enzymes that regulate the metabolite pools at the junction of central metabolism were systematically manipulated to modulate the intracellular NADH. As a result, the engineered strain with the remodeled pathways enabled effective production of *n-*butanol.

## Results and discussion

### Amplification of the pyruvate oxidation pathway

**Fig. 2:**
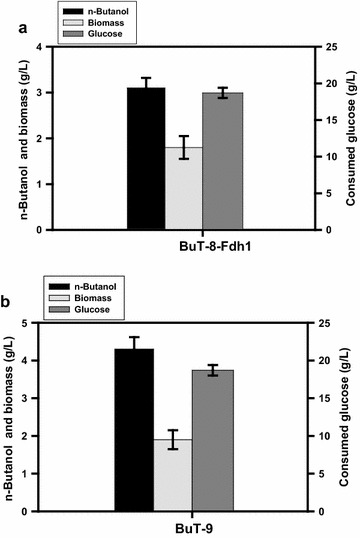
Production of *n-*butanol in strains with the amplification of the pyruvate oxidation pathway. The engineered *E. coli* strains were grown in M9Y medium containing 20 g/L glucose. The fermentations were conducted under the oxygen*-*limited condition for 24 h. The experiments were conducted in triplicate. Keys: **a** the fermentation performance for strain BuT-8-Fdh1; **b** the fermentation performance for strain BuT-9

As shown in Fig. 1, the reductive synthesis of one *n-*butanol from one glucose requires more NADH than that provided in glycolysis. Therefore, the approach of NADH replenishment is expected to favor the fermentative production of *n-*butanol. In this context, the pyruvate node connecting glycolysis and the TCA cycle appears to be a potential target for manipulation. In *E. coli*, pyruvate is oxidized to acetyl-CoA by a reaction mediated by PDH under the aerobic growth and by pyruvate formate-lyase (PFL) under the fermentative growth [4]. Formate is the reduced product of the PFL reaction. Found in other microbes, FDH such as *Candida boidinii fdh* and *Saccharomyces cerevisiaefdh1* catalyzes oxidation of formate to CO_2_ associated with NADH generation [20]. These two genes have been employed in *E. coli* to elevate intracellular NADH, resulting in an increase in *n-*butanol production [17, 21]. Accordingly, *S. cerevisiaefdh1* under the control of the *trc* promoter (Ptrc) without *lacO* was integrated into strain BuT-8. Strain BuT-8 was previously constructed with a CoA-dependent pathway of *n-*butanol consisting of the constitutive λP_L_ promoter (PλP_L_)-driven *hbd*, *crt*, and *adhE2* of *Clostridium acetobutylicum, phaA* of *Cupriavidus necator*, and *ter* of *Treponema denticola* [19]. In addition, the undesired pathways involving endogenous *adhE*, *ldhA*, *pta*, and *frdA* were removed from this strain to curtail carbon waste and conserve NADH. Equipped with *S. cerevisiaefdh1*, the resulting strain BuT-8-Fdh1 produced 3.1 g/L *n-*butanol at 24 h (Fig. 2a). This production titer accounts for a 25 % increase over that for strain BuT-8 (Table 1).

**Table 1:** Summary of the fermentation kinetics for main engineered strains

Strain	Y_B_ (g/L)	P_B_ (g/L/h)	Y_B/G_ (g/g)	NADH (μmol/g cell)	Specific enzyme activity (U/mg protein)			
					PDH	Zwf	Pgl	GltA
BuT-8	2.7^a^	0.11^a^	0.14^a^	42.2	0.7	ND	ND	ND
BuT-9	4.3	0.18	0.22	60.9	1.6	6.1	ND	ND
BuT-10	4.9	0.20	0.25	75.8	18.3	0.5	ND	ND
BuT-12	5.4	0.23	0.27	82.6	ND	ND	6.2	2.2
BuT-14	6.1	0.21	0.31	96.1	ND	ND	ND	1.5

^a^The kinetic data were drawn from the previous report [19]. The development course of producer strains for the *n-*butanol production was shown in Additional file 1: Fig. S1. *U* μmole/min, *Y*_*B*_ the *n-*butanol titer, *ND* not determined

In contrast to PFL, the PDH reaction generates NADH as the reduced product. Therefore, manipulation of the PDH level is expected to alter intracellular NADH. This was conducted by fusion of PλP_L_ with *aceEF* operon to enhance the gene expression in strain BuT-8-Fdh1. To render PDH less sensitive to NADH inhibition, the endogenous *lpdA* (encoding dihydrolipoamide dehydrogenase) was deleted and a mutation site (E354K) in *lpdA* was additionally created [22]. The mutant lpdA* under the control of PλP_L_ was then inserted into strain BuT-8-Fdh1 to obtain strain BuT-9. Consequently, strain BuT-9 exhibited 1.3-fold higher PDH activity and 45 % higher NADH level as compared to strain BuT-8 (Table 1). The accumulated pyruvate in strain BuT-8 was greatly reduced and carbon flux was diverted from the synthetic pathways of byproducts (Table 2). Strain BuT-9 finally produced 4.3 g/L *n-*butanol (Fig. 2b), which accounts for a 60 % increase over that in strain BuT-8 (Table 1).

**Table 2:** Carbon recovery of fermentation products for engineered strains during the oxygen-limited growth on glucose

Strain	Pyruvate	Succinate	Ethanol	Lactate	Acetate	Butyrate	Butanol	Total (%)
BuT-8	20.7	3.5	7.8	2.7	1.5	4.1	21.9	62.2
BuT-9	2.6	2.1	5.2	1.8	1.2	2.7	34.8	50.4
BuT-10	0.01	1.1	5.2	1.5	0.8	2.0	39.6	50.2
BuT-12	Nil	0.8	5.2	1.4	0.7	1.4	43.7	53.2
BuT-14	Nil	0.6	2.6	1.3	0.5	1.4	49.4	55.8

Carbon recovery was calculated as the molar percent of carbon in products per carbon in consumed glucose. Nil, carbon recovery less than 0.01

The similar approach has been previously applied for *n-*butanol production in *E. coli* that carries the CoA-dependent synthetic pathway. By using *C. boidinii fdh*, the best strain in their study showed a 1.3-fold increase in *n-*butanol production [17]. In another work, a 1.6-fold improvement in the production yield was reported for a strain with enhanced PDH [15]. In addition, it was reported that a strain with the optimal activation of PDH exhibited a 12 % improvement in *n-*butanol production [18]. Further improvement of the strain by optimization of *S. cerevisiae fdh1* expression led to a 35.4 % increase in the production titer [18]. These studies were conducted using super-rich TB medium, in contrast to ours which employed M9Y medium. Although displaying a various degree of improvement in the *n-*butanol production, aforementioned studies and ours illustrate the feasibility in manipulating the pyruvate node to modulate intracellular NADH.

### Amplification of the pentose phosphate pathway

**Fig. 3:**
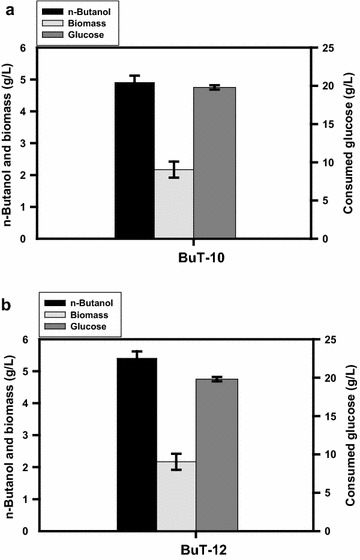
Production of *n-*butanol in strains with the amplification of the PP pathway. The engineered *E. coli* strains were grown in M9Y medium containing 20 g/L glucose. The fermentations were conducted under the oxygen-limited condition for 24 h. The experiments were conducted in triplicate. Keys: **a** the fermentation performance for strain BuT-10; **b** the fermentation performance for strain BuT-12

The glycolytic pathway bifurcates at the glucose-6-phosphate (G6P) node (Fig. 1). With G6P as a starting metabolite, the PP pathway produces precursors for the synthesis of nucleic acids and aromatic amino acids and also provides a major source of NADPH for biosynthesis which is involved in most of the reductive pathways [4]. It is possible to increase NADH availability by manipulation of the G6P node. G6P dehydrogenase (encoded by *zwf*) catalyzes the first step in the PP pathway. Therefore, *zwf* of strain BuT-9 was fused to PλP_L_. In *E. coli*, pyridine nucleotide transhydrogenase (encoded by *udhA*) functions to interconvert NADPH and NADH [23]. Therefore, strain BuT-10 was obtained by further fusion *udhA* of strain BuT-9 with PλP_L_ to enhance its expression. As compared to strain BuT-9, strain BuT-10 displayed twofold higher Zwf activity and a 10 % increase in the *n-*butanol production (4.9 g/L) (Table 1; Fig. 3a).

The developed strain is derived from strain BL21 which lacks *pgl* [24], a gene encoding lactonase that is responsible for the reaction following Zwf in the PP pathway. It is likely that *yieK* with an annotated function of Pgl is functioning in *E. coli* B strain but less active. Therefore, the carbon flux channeled into the PP pathway by elevated Zwf may be limited at the Pgl-mediated reaction step. To address this issue, the PλP_L_-driven *pgl* from *E. coli* K-12 strain was re-introduced into strain BuT-10. Finally, the resulting strain (BuT-12) enabled production of 5.4 g/L *n-*butanol (Fig. 3b). As compared to strain BuT-10, strain BuT-12 displayed a tenfold higher Pgl activity, a 36 % more NADH, and a 25.6 % improvement in the *n-*butanol production (Table 1). The approach by enhancing the PP pathway results in more NADH production, which drives more acetyl-CoA into the synthetic pathway of *n-*butanol. This is supported by the observed decrease in pyruvate and succinate (Table 2).

It is apparent that redistribution of carbon flux in glycolysis and the PP pathway can greatly affect intracellular NADH level. Notice that entry of one glucose into the oxidative PP pathway generates two reducing equivalents but wastes one CO_2_. Nevertheless, strain BuT-12 which is manipulated at the pyruvate and G6P nodes displays a 96 % increase in the NADH level and doubles the *n-*butanol production as compared to strain BuT-8 (Table 1).

### Rerouting catabolic pathways of glucose

**Fig. 4:**
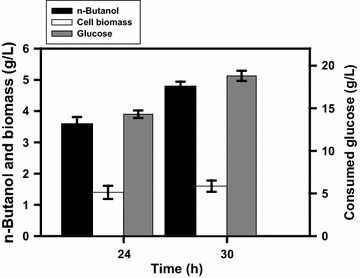
Production of *n-*butanol in the strain with the glucose catabolism via the PP pathway. Strain BuT-13 grown in M9Y medium containing 20 g/L glucose. The fermentations were conducted under the oxygen-limited condition for 30 h. The experiments were conducted in triplicate

According to the central metabolism of *E. coli* (Fig. 1), glucose catabolism proceeding via the PP pathway generates 85 % more reducing power per gram mole of glucose than that via glycolysis. It seems useful to increase intracellular NADH by diverting the glycolytic flux to the PP pathway. Phosphoglucose isomerase (encoded by *pgi*) is responsible for isomerization of G6P, and its inactivation makes the PP pathway the primary route of glucose catabolism [25]. Therefore, strain BuT-13 was obtained by deletion of *pgi* in strain BuT-12. In comparison with strain BuT-12, strain BuT-13 grew poorly (0.31/h vs. 0.5/h), while it exhibited a 32 % and 30 % decrease in biomass yield and glucose utilization, respectively (Fig. 4). After fermentation for 30 h, strain BuT-13 was unable to consume all glucose and produced less *n-*butanol (4.6 g/L).

Improving NADPH availability was realized by a strain deprived of *pgi*, whereas the strain showed a 47 % decrease in the specific growth rate [26]. The severe growth defect resulting from *pgi* knockout is attributed to a surplus of NADPH, which in turn perturbs the physiological state of cells [27]. Interestingly, an elevated level of either UdhA or Zwf can lead to growth recovery of the *pgi*-deficient strain by 25 and 68 %, respectively [27, 28]. Strain BuT-13 is still afflicted by the growth defect although it exhibits higher UdhA and Zwf activities and harbors the *n-*butanol synthetic pathway that consumes the reducing equivalent. The result implies the presence of an imbalanced redox state in the strain.

**Fig. 5:**
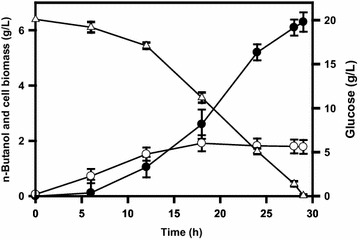
Time course of *n-*butanol production in the strain with the limited TCA cycle. Strain BuT-14 grown in M9Y medium containing 20 g/L glucose. The fermentations were conducted under the oxygen-limited condition. The experiments were conducted in triplicate

In response to the oxygen tension, the TCA cycle operates as either an oxidative pathway or a reductive pathway associated with production of various levels of reducing equivalents [4]. Citrate synthase (encoded by *gltA*) catalyzes the first committed step in the TCA cycle (Fig. 1). The approach to divert carbon flux from the TCA cycle by lowering the GltA activity is expected to conserve acetyl-CoA (the precursor of *n-*butanol ) and modulate production of reducing equivalents. It may be helpful to ameliorate the negative impact on the strain imposed by null *pgi*. This was carried out by replacement of the *gltA* cognate promoter P2 with *lacO* site in strain BuT-13. The resulting strain BuT-14 was then cultured and examined for its fermentation performance. Consequently, strain BuT-14 grew almost normally (ca. 0.46/h), and its biomass yield was comparable to strain BuT-12. All fermentation byproducts were significantly reduced in strain BuT-14 which consequently produced 6.1 g/L *n-*butanol at 29 h (Table 2; Fig. 5). As expected, strain BuT-14 exhibited 32 % less GltA activity and 16 % more NADH as compared to strain BuT-12. Direction of carbon flux through the PP pathway by *pgi* deletion increases the reducing equivalent, whereas prevention of carbon flux from entering the TCA cycle by lowering GltA reduces the reducing equivalent. It leads to a net outcome of a moderate increase in NADH, which suffices the need for the synthesis of *n-*butanol. As a result, a redox-balanced state is established in strain BuT-14 that recovers from the null *pgi*-induced growth defect. It was intriguing to learn the response of this strain to the act of further lowering the GltA activity. Strain BuT-14-A was thus obtained by integration of *lacI*^Q^ into strain BuT-14. Consequently, strain BuT-14-A displayed 50 % less GltA activity than strain BuT-12 (Table 1). The strain exhibited a poor growth and consumed only 40 % glucose associated with the *n-*butanol production of 1.8 g/L at 30 h (data not shown). It was reported that the growth of *E. coli* on glucose remains unaffected by a 90 % decrease in the GltA activity [29]. In contrast, the growth of the *n-*butanol-producing strain that lacks *pgi* on glucose is closely linked to the GltA activity. By modulation of the GltA activity, the producer strain enables recovery from the growth defect caused by null *pgi*. It is likely that the alteration of the GltA activity perturbs the intracellular redox state of the strain. Apparently, the engineered strain displays high susceptibility to the intracellular redox state and optimal adjustment of GltA activity is necessary to ensure the superior performance of the strain.

Recruitment of FDH and enhanced expression of PDH have been proposed to increase NADH availability favoring the *n-*butanol production in *E. coli*. In one study, the approach by recruiting FDH achieved the *n-*butanol productivity (P_B_) of 0.2 g/L/h and the conversion yield on glucose (Y_B/G_) of 0.36 g/g [17]. An alternative study by manipulating PDH reported P_B_ of 0.065 g/L/h and Y_B/G_ of 0.19 g/g [15]. In addition, P_B_ of 0.26 g/L/h and Y_B/G_ of 0.27 g/g were obtained by optimization of both FDH and PDH activity in a recent work [18]. All these studies were conducted with super-rich TB medium, and plasmids were employed for the episomal expression of multiple target genes to increase their expression levels, which is important to obtain the observed effect. Therefore, the discrepancy in the fermentation production by these reports is likely attributed to various expression levels of the cloned genes. However, plasmid maintenance is known to impose a metabolic burden on cells, thus resulting in a reduced growth rate and perturbation of the cell physiology [30]. A metabolic load is additionally imposed on *E. coli* after the forced expression of the plasmid-borne genes, which suppresses the primary carbon and energy metabolism of cells [31]. The use of TB medium (12 g/L tryptone, 24 g/L yeast extract, 2.31 g/L KH_2_PO_4_, 12.54 g/L KHPO_4_, 4 mL/L glycerol) is expected to improve the cell growth under the anaerobic condition and helps to ameliorate the plasmid-induced stress on cells. It should be noted that components other than glucose in the TB medium could contribute 15 % of the *n-*butanol production according to the previous study [17]. Therefore, these reported yields based on glucose may be re-estimated. In contrast, the engineered strains were free of plasmids and grown on the M9Y medium with glucose in this study. First, the starter strain BuT-8 displayed P_B_ of 0.11 g/L/h and Y_B/G_ of 0.14 g/g (Table 1). With both enhanced FDH and PDH, strain BuT-9 achieved P_B_ of 0.18 g/L/h and Y_B/G_ of 0.22 g/g. Finally, strain BuT-14 was developed by rerouting of the glycolytic flux through the PP pathway and further reducing GltA activity in the TCA cycle. It exhibited 2.3-fold higher NADH and *n-*butanol production titer than the starter strain BuT-8. Notice that *n-*butanol was not produced for strain BuT-14 grown on M9Y medium without glucose (data not shown). Therefore, the *n-*butanol production in the strain comes merely from glucose. As a result, strain BuT-14 displayed P_B_ of 0.21 g/L/h and Y_B/G_ of 0.31 g/g. In theory, 0.85 mol instead of 1 mol *n-*butanol per mole glucose is produced in the glucose catabolism via the PP pathway due to CO_2_ loss, which leads to the theoretical yield of *n-*butanol as 0.35 g/g. Accordingly, *n-*butanol remains the main product for strain BuT-14 (Table 2) with Y_B/G_ reaching 89 % of the theoretical. It is feasible to recycle CO_2_ in *E. coli* with the expression of *Synechococcus* ribulose-1,5-bisphosphate carboxylase/oxygenase (Rubisco) and phosphoribulokinase [32]. The issue of increasing Y_B/G_ may be addressed by recruitment of the Rubisco-based CO_2_ recycling system in strain BuT-14. Overall, it indicates that our proposed approach is effective for production of the highly reduced product without the need for a super-rich medium.

## Conclusions

The intracellular redox state in microbes is recognized as a key factor determining the production efficiency of fermentation products. The current work illustrates that pyruvate, G6P, and acetyl-CoA at the junction of the central catabolism are flexible for engineering. It is known that the enzymes, such as PDH and GltA, involved in the use of these metabolite nodes are subjected to the physiological control mediated by the effector metabolites [4]. Nevertheless, genetic manipulation of these metabolite nodes can lead to the redistribution of carbon flux, which in turn alters the cellular redox state. In principle, these metabolite nodes can be individually or coordinately modulated to fulfill the intracellular need for high production of reduced products of interest.

## Methods

### Bacterial culturing

The method for bacterial culturing under the oxygen*-*limited condition essentially followed the previous report [19]. The seeding cultures were prepared by growing *E. coli* strains on Luria–Bertani medium [33] with 2 g/L glucose overnight. The cell density was measured turbidimetrically at 550 nm (OD_550_). The overnight culture was inoculated into capped Erlenmeyer flasks (125 mL) containing 50 mL M9Y medium (6 g/L Na_2_HPO_4_, 3 g/L KH_2_PO_4_, 0.5 g/L NaCl, 1 g/L NH_4_Cl, 1 mM MgSO_4_, 0.1 mM CaCl_2_, 10 mg/L vitamin B1, 5 g/L yeast extract) with 20 g/L glucose to attain the initial cell density at OD_550_ of 0.2. The shake`flask cultures were maintained in an orbital shaker set at 100 rpm.

### Strain construction

The strains and primers applied in this study are listed in Table 3. Genomic insertion of *fdh1* into strain BuT-8 was constructed previously [34]. In brief, Ptrc-driven *fdh1* of *S. cerevisiae* was amplified from plasmid pTrc-Fdh1 [21] by PCR with primers RC12171/RC12314. After digestion with *Bam*HI, the PCR DNA was spliced into *Bam*HI-*Nru*I of plasmid pP21-Km to obtain plasmid pP21-Fdh1. Based on our reported methods, the DNA containing Ptrc-*fdh1* was then integrated into *E. coli* using plasmid pP21-Fdh1, and the inserted kanamycin-resistant marker in the strain was later removed [35]. Strain BuT-8 with *fdh1* was renamed BuT-8-Fdh1.

**Table 3:** The strains and primers applied in this study

	Characteristics	Source
Strains		
BuT-8	Δ*frdA* ɸ80*attB*:: PλP_L_-*crt*	
	Δ*adhE*::ɸ80*attB*::PλP_L_-*pha*-*hbd*	
	Δ*ldhA*::λ*attB*::PλP_L_-*adhE2*	19
BuT-8-Fdh1	as P21*attB*:: Ptac-*fdh1*	34
BuT-9	as But-8-Fdh ∆*lpdA* λ*attB*::PλP_L_-*lpdA* ^*^ PλP_L_-*aceEF*	This study
BuT-10	as But-9 PλP_L_-*zwf* PλP_L_-*UdhA*	This study
BuT-12	as But-10 Δ*atoD*::PλP_L_-*pgl*	This study
BuT-13	as But-12 ∆*pgi*	This study
BuT-14	as But-13 *lacO*-*gltA*	This study
Primers		
RC10178	ATAAGGATCCATATCTAACACCGTGCGTG	
RC11210	CACACCATATGTTAGAATTCATTACCTTCG	
RC11403	TTTGCGGTACCAAGCCCTTTGCAAATTGC	
RC11404	CAGCAGAGCTCGAATGGATCGCGTTATC	
RC11405	AGAATCATATGGCGGTAACGCAAACAG	
RC11406	CTTAAGGATCCTAACCCGGTACTTAAGCCAG	
RC11407	CGTAAGGTACCTGACGCATGCGCGTTTG	
RC11408	ACTTAGAGCTCTAAATGCGGCTTCCACCAG	
RC11409	GCCCTCATATGCCACATTCCTACGATTAC	
RC11410	TGTTCGGATCCATAAAAGCAACAGAATGGTAAC	
RC11417	CCAAGCCCTTTGCAAATTGC	
RC11418	CTCGAATGGATCGCGTTATC	
RC11419	CCTGACGCATGCGCGTTTG	
RC11420	CTAAATGCGGCTTCCACCAG	
RC12058	AATAACATATGTCAGAACGTTTCCCAAATG	
RC12059	CTATCTCTAGACGTTGAGTTTTCTGGAACC	
RC12060	CCAGTTCGAGGTCTTTTTTCG	
RC12085	TATGGGGTACCAGTTCGAGGTCTTTTTTCG	
RC12086	CAATGGAGCTCTGCTTCATCTGCTAAGG	
RC12154	GCGATATCGTCGGTCAACC	
RC12155	TGAGAAGCTTCAGTCCGCATCACCAGAG	
RC12171	GCAAGCTTATTTCTTCTGTCCATAAGC	
RC12215	GTCCATCGCCTATACCAAACCAGAAGTTGCATG	
RC12216	CATGCAACTTCTGGTTTGGTATAGGCGATGGAC	
RC12288	AACTGCTCGAGTTACTTCTTCTTCGCTTTCG	
RC12289	AAGTGGATCCATACCCGTCGTCTTTCAGG	
RC12290	CCATGAGCTCGGCTTTTTTCTGGTAATCTC	
RC12314	TCTGGGGATCCTTCTGAAATGAGCTGTTGAC	
RC12331	ACTCTCGAATTCTGGTCGTCCTATCGCTTC	
RC13001	TTGAATTCCGCCTTTAAAGATCGCCATG	
RC13034	CATCTCACCAGATATCATGC	
RC13035	AATCGGAGCTCGAAAGTGAACTGTTTGG	
RC13195	ATCTTCCCGGGCGGAATTCATTACCGTTC	
RC13196	GAAATTGTTATCCGCTCACAATTCCGGGTACCCAATTC	
RC13197	CAGCAAAATACCTTCATCACC	
RC13198	TTCAGGGGAAGAGAGGCTG	
RC13199	TCAATGGGCCCACACTGTTACATAAGTTAATC	
RC13200	TTAATGTCGACGATTGCTAAGTACTTGATTCG	
RC13201	GGTACCCAGAAGCCACAG	
RC13292	ATCCCGGGAAGCAAACAGTTTATATCGC	
RC13293	ATCTCGAGTTAGTGTGCGTTAACCACCAC	
RC14025	GAGGAATTCTGTAGGCTGGAGCTGCTTC	
RC14026	AACGGTCGACATGGGAATTAGCCATGG	

Plasmid pMCS-lpdA was obtained by PCR-amplification of *lpdA* from strain BL21 with primers RC12154/RC12155 and subsequent incorporation into *Nde*I-*Xho*I of plasmid pMCS-5. The E354K mutation was introduced into *lpdA* on plasmid pMSC-lpdA by the site-directed mutagenesis using primers RC12215/RC12216. The mutant *lpdA* (lpdA*) was confirmed by DNA sequencing and removed from plasmid pMCS-lpdA by *Nde*I-*Xho*I. The recovered lpdA* was subcloned into plasmid pLoxKm-PR [36] which carries a cassette of PλP_L_ fused with LE*-*kan*-RE* (LE*-*kan*-RE*-PλP_L_). The resulting plasmid pLoxKm-lpdA* contains the LE*-*kan*-RE*-PλP_L_-regulated lpdA* (LE*-*kan*-RE*-PλP_L_-lpdA*). Meanwhile, the upstream region of *lpdA* was amplified by PCR with primers RC12289/RC12290 and spliced into *Bam*HI-*Sac*I of plasmid pBluescript to produce plasmid pBlue-ac. The DNA containing LE*-*kan*-RE*-PλP_L_-lpdA* was recovered from plasmid pLoxKm-lpdA* by *Bam*HI-*Xho*I and then incorporated into plasmid pBlue-ac to give plasmid pBlue-ac/lpdA*. In addition, PCR was conducted on plasmid pBlue-ac/lpdA* with primers RC11210/RC12331. The PCR DNA was digested with *Eco*RI and self-ligated to give plasmid pBlue-Ac-lpd, which carries *lpdA* interrupted with LE*-*kan*-RE*. To knockout *lpdA*, the truncated *lpdA* was amplified from plasmid pBlue-Ac-lpd by PCR with primers RC12288/RC12290 and electroporated into *E. coli* following our protocol. Finally, the DNA containing PλP_L_-lpdA* was amplified from plasmid pBlue-ac/lpdA* by PCR with primers RC10178/RC12288 and then restricted by *Bam*HI. Plasmid pLam-LpdA* was obtained by incorporation of the PCR DNA into *Bam*HI-*Eco*RV of plasmid pLam-Crt [36]. Similarly, the DNA containing PλP_L_-lpdA* was integrated into *E. coli* followed by removal of the inserted marker [21].

To enhance the expression of endogenous genes, PλP_L_ was placed in front of the structural genes with their cognate promoters intact. This was carried out as follows: First, the upstream region and 5′-end structural regions of *zwf*, *udh, and aceE* were amplified from strain BL21 by PCR with primers RC11403/RC11404, RC11407/RC11408, and RC12085/RC12086, respectively. Each PCR DNA was digested by *Kpn*I-*Sac*I and incorporated into plasmid pBluescript to obtain plasmid pBlue-zwf, pBlue-udhA, and pBlue-aceE. Secondly, the *Nde*I-*Bam*HI site was introduced into plasmid pBlue-zwf and pBlue-udhA by PCR with primers RC11405/RC11406 and RC11409/RC11410 while the *Nde*I-*Xba*I site into plasmid pBlue-aceE with primers RC12058/RC12059. The LE*-*kan*-RE*-PλP_L_ cassette was recovered from plasmid pLoxKm-PR by *Nde*I-*Bam*HI or *Nde*I-*Xba*I digestion and then incorporated into plasmid pBlue-zwf, pBlue-udhA, and pBlue-aceE to obtain plasmid pPR-zwf, pPR-udhA, and pPR-aceE. Finally, the PCR DNAs were amplified from plasmid pPR-zwf, pPR-udhA, and pPR-aceE with primers RC11417/RC11418, RC11419/RC11420, and RC12060/RC12086, respectively. These passenger DNAs were individually integrated into the strain by electroporation according to the reported method [21]. The associated marker was finally rescued.

To obtain *pgl*, the gene was amplified from strain MG1655 with primers RC13292/RC13293. After cleavage by *Eco*RV-*Sac*I, the PCR DNA and plasmid pBluescript were spliced together to give plasmid pBlue-pgl. The *pgl*-containing DNA was recovered by *Sma*I-*Xho*I and incorporated into plasmid pLoxKm-PL. The construction resulted in plasmid pSPL-pgl, which fuses LE*-*kan*-RE*-PλP_L_ with *pgl*. The LE*-*kan*-RE*-PλP_L_-*pgl* DNA was amplified by PCR with primers RC13001/RC13293. Plasmid pAto-pgl was obtained by incorporation of the PCR DNA into *Eco*RI-*Nru*I of plasmid pSPL-atoD [36]. Similarly, the passenger DNA was amplified from plasmid pSPL-atoD with primers RC13034/RC13035 and then electroporated to the strain. Later removal of the inserted marker was carried out.

To modulate the *gltA* expression, its P2 promoter was replaced with *lacO*. This was done in several steps. First, *lacO* was created in plasmid pLoxKm-PR by PCR with primers RC13195/RC13196. After cleavage by *Sma*I, the PCR DNA was self-ligated to produce plasmid pLoxCm-LacO, which carries the fusion of LE*-*kan*-RE*-*lacO*. Secondly, the DNA containing the upstream region and 5′-end structural sequence of *gltA* was amplified from strain BL21 by PCR with primers RC13197/RC13198. Plasmid pBlue-GltA was generated by incorporation of the PCR DNA into *Kpn*I-*Sma*I of plasmid pBluescript. Moreover, the *Apa*I-*Sal*I site was introduced into plasmid pBlue-GltA by PCR with primers RC13199/RC13200. The LE*-*kan*-RE*-*lacO* cassette was recovered from plasmid pLoxCm-LacO by *Apa*I-*Sal*I and incorporated into plasmid pBlue-GltA to give plasmid pBlue-GltO. Finally, the FRT-*Cm*-FRT cassette was amplified from plasmid pKD3 by PCR with primers RC14025/RC14026. The LE*-*kan*-RE* cassette was replaced by FRT-*Cm*-FRT by incorporation of the PCR DNA into *EcoR*I-*Sal*I of plasmid pBlue-gltO, leading to plasmid pB-gltO-Cm. The passenger DNA was amplified from plasmid pB-gltO-Cm with primers RC13197/RC13201 and then electroporated to the strain in a similar way.

### Analytical method

The analytical method essentially followed our reported protocol [19]. Glucose was measured by high-performance liquid chromatography (HPLC) with Reflective Index RID-10A (Shimadzu, Japan). *n-*Butanol was determined by Gas Chromatograph Trace 1300 (Thermo Scientific, USA).

The intracellular NADH level was measured by using the fluorescent NAD/NADH detection kit (Cell Technology, USA). The assay procedure exactly followed the manufacturer’s instruction. In brief, bacterial cultures were harvested by centrifugation and the cell pellets were resuspended in 200 µL NADH extraction buffer plus 200 µL lysis buffer. The mixture was kept at 60 °C for 20 min. After centrifugation, the supernatant was recovered and mixed with the reaction reagent for the measurement. The reaction was kept in dark at room temperature for 1 h. The NADH level was then measured with the excitation at 530–570 nm and emission at 590–600 nm.

### Enzyme activity assay

Bacterial cultures were harvested by centrifugation and the cell pellets were resuspended in 1 mL solution buffer. Cells were disrupted by sonication after centrifugation. The supernatant was saved as the cell-free extract (CFX). The total protein content in CFX was determined using Bio-Rad protein assay kit. The activity of pyruvate dehydrogenase was determined by monitoring the reduction of NAD^+^ at 340 nm at room temperature according to the previous report [15]. The reaction solution (1 mL) contains 50 mM potassium phosphate (pH 7.9), 5 mM sodium pyruvate, 1.3 mM CoA, 2 mM NAD^+^, 0.5 mM thiamine pyrophosphate, and 5 mM MgCl_2_. To start the reaction, 100 µL CFX was added to the solution. The activity of glucose-6-phosphate dehydrogenase was determined by monitoring the reduction of NADP^+^ at 340 nm following the reported protocol [37]. The reaction solution (1 mL) is composed of 2 mM glucose-6-phosphate, 0.67 mM NADP^+^, 10 mM MgCl_2,_ and 50 mM Tris–HCl (pH 7.5). The reaction was initiated by adding 100 µL CFX to the solution at 30 °C. The method for measuring the activity of lactonase was similar to the glucose-6-phosphate dehydrogenase activity assay [38]. The reaction solution comprises 50 μM glucose-6-phosphate, 0.5 mM NADP^+^, 50 mM Tris–HCl, 10 mM MgCl_2_, and 50 mM Tris–HCl (pH 7.5). In addition, the citrate synthase activity was measured as reported previously [29]. The composition of the assay solution includes 0.1 mM acetyl-CoA, 0.5 mM oxaloacetate, 0.2 mM 5′5-dithiobis-(2-nitrobenzoic acid), and 50 mM Tris–HCl (pH 7.5).

## Additional file

10.1186/s13068-016-0467-4 The development course of *E. coli* strains for the fermentative production of n-butanol.

## Abbreviations

PP: pentose phosphate
G6P: glucose-6-phosphate
PDH: pyruvate dehydrogenase
FDH: formate dehydrogenase
TCA: tricarboxylic acid
PFL: pyruvate formate-lyase
HPLC: high-performance liquid chromatography
CFX: cell-free extract
Ace: acetate
EtOH: ethanol
F6P: fructose-6-phosphate
Lac: lactate
For: formate
Glc: glucose
OAA: oxaloacetate
PEP: phosphoenolpyruvate
3-PGA: 3-phosphoglyceraldehyde
Pyr: pyruvate
Suc: succinate
