# Supplementary material for: Systematic engineering of the central metabolism in Escherichia coli for effective production of n-butanol
Source: Biotechnol Biofuels. 2016 Mar 18;9:69. doi: 10.1186/s13068-016-0467-4 (PMC4799531; doi:10.1186/s13068-016-0467-4)
Supplement: Supplementary file 1 — 10.1186/s13068-016-0467-4 The development course of E. coli strains for the fermentative production of n-butanol. [file 13068_2016_467_MOESM1_ESM.doc]

**Supplementary Material**

**for**

**Systematic engineering of the central metabolism in *Escherichia coli* for effective production of n-butanol**

Mukesh Saini,^1^ Si-Yu Li,^2^  Ze Win Wang,^1^ Chung-Jen Chiang,^3*^ Yun-Peng Chao,^1,4,5*^

^1^Department of Chemical Engineering, Feng Chia University

100 Wenhwa Road, Taichung 40724, Taiwan

^2^Department of Chemical Engineering, National Chung Hsing University, Taichung 402, Taiwan

^3^ Department of Medical Laboratory Science and Biotechnology, China Medical University, No. 91, Hsueh-Shih Road, Taichung 40402, Taiwan

^4^Department of Health and Nutrition Biotechnology, Asia University, Taichung 41354, Taiwan

^5^Department of Medical Research, China Medical University Hospital, Taichung 40447, Taiwan

Fig. S1. The development course of *E. coli* strains for the fermentative production of n-butanol. Shown were the production titer obtained by each engineered strain. The data were taken from Figs. 2A, 2B, 3A, 3B, and 5.
